# 

**Published:** 1905-03

**Authors:** 


					﻿Sixtieth Year.
MARCH. 1905.
BUFFALO,
MEDICAL JOURNAL
VOL. LX.
Whole Number,
DCC.
New Series.
VOL. XLIV
> < y
Established 1845 by AUSTIN FLINT, M. I).
EDITOR :
WILLIAM WARREN POTTER, M. D.
ASSISTANT EDITORS:
WILLIAM C. KRAUSS, M. D.
NELSON W. WILSON, M. D.
ASSOCIATE EDITORS:
JAMES WRIGHT PUTNAM, M. D.
JOHN PARMENTER, M. D.
HARVEY R. GAYLORD, M. D.
ERNEST WENDE, M. D.
JOHN A. MILLER, Ph. D.
MAUD J. FRYE, M. D.
For terms and other information relating to the Journal, see page ▼!.
				

